# Relationship between c-reactive protein to albumin ratio and coronary artery calcium score and CAD-RADS scores with coronary computed tomography angiography

**DOI:** 10.3906/sag-2103-104

**Published:** 2021-10-21

**Authors:** Mehmet Akif ERDÖL, Kadriye GAYRETLİ YAYLA

**Affiliations:** 1 Department of Cardiology, Faculty of Medicine, Ankara City Hospital, University of Health Sciences, Ankara Turkey; 2 Department of Cardiology, Faculty of Medicine, Dr. Abdurrahman Yurtaslan Ankara Onkoloji Education and Research Hospital, University of Health Sciences, Ankara Turkey

**Keywords:** Coronary calcium score, CAD-RADS score, coronary computed tomography angiography, serum C-reactive protein to albumin ratio

## Abstract

**Background/aim:**

C-reactive protein (CRP) to albumin ratio (CAR) is predictive marker of systemic inflammatory state in atherosclerotic coronary diseases when compared to predictive value of these two markers separately. We aimed to evaluate the relationship between CAR and the coronary artery calcium (CAC) score, Coronary Artery Disease-Reporting and Data System (CAD-RADS) score in patients’ unknown diagnosis of coronary artery disease (CAD) underwent coronary CTA (Computed Tomography Angiography) and were classified by CAD-RADS scores.

**Materials and methods:**

A total of 187 patients consecutively referred for the evaluation of their chest pain underwent coronary CTA were included retrospectively.

**Results:**

CRP, CAR, and CAD-RADS scores were higher in patients with CAC score > 400 than the other groups (p < 0.001). We found positive correlation between CAR and CAC score (r= 0.384, p < 0.001), and also there was a positive correlation between CAR and CAD-RADS score (r= 0.462, p < 0.001). Multivariate logistic regression analyses showed that low density lipoprotein cholesterol (LDL-C), CAD-RADS score, and CAR were independent predictors of CAC score (p < 0.05).

**Conclusion:**

Higher CAR can be a predictive marker of atherosclerosis and CAD. CAR may be useful in the management of patients before invasive coronary angiography. Further studies are needed to clarify the pathophysiologic role of CAR in patients with atherosclerotic coronary heart diease.

## 1. Introduction

Coronary computed tomography angiography (CCTA) is an anatomical noninvasive evaluation of coronary arteries and provides high diagnostic accuracy for the exclusion and detection of obstructive coronary artery disease (CAD) (≥50% stenosis) compared to invasive coronary angiography [1]. The use of CCTA has been increasing in recent years due to the fact that it is a noninvasive examination, its high negative predictive value in excluding CAD, and recent technological advancement [2]. European Society of Cardiology Chronic Coronary Syndromes guidelines recommend CCTA as an appropriate test to exclude obstructive CAD in low-to intermediate-risk patients with chest pain or chest pain equivalent.[3] Some studies have shown that obstructive CAD in CCTA is related with adverse outcomes in comparison to nonobstructive or non-CAD [4, 5]. The Coronary Artery Disease Reporting and Data System (CAD-RADS), which aims to classify and standardize the severity of CAD in CCTA, has been recently published [6, 7].

The results of coronary artery calcium (CAC) score calculated by CCTA are obtained quickly and require a little time for preparation. Patients who admitted with chest pain can be discharged with low CAC calculated in CCTA on the same day. In addition, the results obtained by CCTA can be used as a reference value for subsequent follow-ups. Zero calcium score is a strong negative risk predictor for coronary artery disease, with a negative predictive value of 99%, sensitivity 91%, and specificity 64% [8, 9].

C- reactive protein (CRP) is a highly sensitive acute phase reactant and is produced promptly and in large quantities by the liver due to various inflammatory stimuli with intermediary cytokines such as interleukin (IL)-6 [10, 11]. In addition, CRP can be formed by other tissues, including kidneys, adipocytes, arteries, and vascular smooth muscle cells [11]. Albumin is a negative acute-phase protein. Hypoalbuminemia is related to the intensity of the infection triggered inflammatory response in critically ill patients [12]. Recently, CRP to albumin ratio (CAR) is used as a predictive marker of systemic inflammatory state in atherosclerotic coronary diseases when compared to predictive value of these two markers separately [13–15]. In this study, we aimed to evaluate the relationship between CAR and CAC, CAD-RADS score in patients unknown diagnosis of CAD who underwent CCTA and were classified by CAD-RADS scores,

## 2. Methods

The study was conducted at Ankara City Hospital and reviewed retrospectively between January and December 2020. A total of 187 patients who consecutively admitted to our hospital with chest pain underwent CCTA were included in the study. Patients were stratified into very low, low - moderate and high risk groups according to the CAC score (0, 1-400 and> 400, respectively) defined by the American College of Cardiology Foundation/American Heart Association [16]. Exclusion criteria were determined as existence of acute coronary syndrome, history of cardiovascular diseases, decompensated heart failure, severe valve disease, malignancy, hepatic or renal malfunction, acute or chronic infection, anemia or hematologic disease, autoimmune disease or those receiving immunosuppressive therapy, and chronic obstructive lung disease. The study was conducted in accordance with the principles stated in the Helsinki Declaration and was approved by the local ethics committee. (Ethics committee no: E1-21-1567) 

Baseline clinical demographic characteristics were evaluated. Hypertension (HTN) was described as documentation of a systolic blood pressure of ≥140 mmHg and/or a diastolic blood pressure of ≥90 mmHg in at least two measurements or active use of any antihypertensive agent. Diabetes mellitus (DM) was defined as a fasting plasma glucose level over 126 mg/dL or glucose level over 200 mg/dL at any measurement or active use of an antidiabetic agent.

Blood samples were taken in the morning from patients who had fasted overnight before CCTA. Coulter Counter LH Series (Beckman Coulter Inc, Hialeah, Florida) was used for complete blood count (CBC) analysis. The ejection fraction was calculated by the modified Simpson method using a Philips Affiniti 50 Echocardiography device and a 2–4 mhz transducer (Philips, Healthcare, Andover, The Netherlands). The albumin and CRP levels were obtained using a Roche Diagnostics Cobas 8000 c502 analyzer (Indianapolis, USA). The CAR was calculated as the ratio of CRP to the albumin level.

### 2.1. Coronary computed tomography angiography and image analysis 

A 512-row multi detector computed tomography (MDCT) scanner (Revolution; GE Healthcare, Milwaukee, WI, USA) was used according to the standard spiral protocol to obtain a volume data set of the heart. CAC was measured by the Agatston method (Revolution; GE Healthcare, Milwaukee, WI, USA). Nitroglycerin spray was administered sublingually immediately prior to each scan. Oral beta-blocker was started 3 days before CCTA, and if the heart rate was >70 beats per minute during the procedure, additional intravenous beta blockers were administered. CAC scoring was evaluated using the GE Healthcare Smartscore program and Agatston method by determining the total score of calcium plaques above 130 Hounsfield units (HU) density threshold in the coronary arteries. All plaques on the coronary arteries were evaluated manually. The severity of coronary artery stenosis was evaluated by visual estimation.

### 2.2. CAD-RADS classification 

The CAD-RADS classification system was applied to each patient representing the highest grade coronary artery stenosis documented by the CCTA. A summary of the CAD-RADS classification is shown in Table 1 (Adapted from Cury et al. [17]).

**Table 1:** CAD-RADS classification.

Classification	Definition	Further investigation
0	Absence of CAD	None
1	Minimal stenosis or plaque without stenosis (1%–24%)	None
2	Mild stenosis (25%–49%)	None
3	Moderate stenosis (50%–69%)	Consider functional assessment
4	4A: 70%–99% stenosis4B: LM >50% or 3 vessel obstructive disease (≥70%)	4A: Consider functional assessment or ICA4B: ICA recommended
5	Total occlusion (100%)	Consider ICA and/or viability assessment

CAD, coronary artery disease; ICA, invasive coronary angiography; LM, left main.

### 2.3. Statistical analysis

The SPSS 21.0 Statistical Package Program for Windows (SPSS Inc., Chicago, IL, USA) was used for statistical analysis. The normality of the distribution was evaluated with the Kolmogorov–Smirnov test.  Quantitative variables with a normal distribution were specified as the mean ± standard deviation and variables with non-normal distribution were shown as median (interquartile range), categorical variables were calculated as percentages. One-way ANOVA test was chosen to demonstrate the differences between the groups in continuous numeric parameters with normal distribution. Tukey’s test was selected for the post-hoc analysis. The Kruskal–Wallis test was chosen to compare more than two groups with no normal distribution. In order to determine the difference between which groups, the Mann–Whitney U test was applied. Chi-square test was used for categorical variables. The relationship between CAD-RADS score, CAR AND CAC score was calculated by Spearman’s correlation analysis. Binary Logistic regression analysis was used to determine the odds ratio (ORs) and 95% confidence intervals (CIs) of possible confounding factors in CAC Score (>400). The univariate regression model was used for possible confounding factors and confounders with a p value <0.1 were included in the multivariate analysis. A p value of <0.05 was considered statistically significant.

## 3. Results

Baseline characteristics and laboratory parameters of the study groups according to the CAC Score are shown in Table 2. The rate of HTN, DM were differed into three groups. There were significantly higher rate of HTN patients in the CAC score > 400 group than the CAC score = 0 group (p = 0.028). There were significant differences in the frequency of DM patients between all groups (p < 0.001 among all groups). Also, level of serum albumin, alanine aminotransferase, hemoglobin A1c, creatinine, total cholesterol, low density lipoprotein cholesterol (LDL-C), and high-density lipoprotein cholesterol (HDL-C) were significantly differed in three groups (p < 0.05). CRP, CAR and CAR-RADS score was higher in patients with CAC Score > 400 than the other groups (p < 0.001). There was significant positive correlation between CAR and CAC score (r = 0.384, p < 0.001). In addition, a positive correlation was found between CAR and CAD-RADS score (r = 0.462, p < 0.001). Since, CAD-RADS score ≥ 4 is associated with severe coronary artery stenosis. Therefore, the participants were divided into 2 groups according to the CAD-RADS score < 4 and CAD-RADS score ≥ 4. CAR [0.66 (0.30–1.09) vs. 1.73 (1.01–3.33); p < 0.001] and CAC score [10 (0–107) vs. 477 (110–1111); p < 0.001] was significantly higher in patients with CAD-RADS score ≥ 4 (Table 3).

**Table 2:** Characteristics and laboratory parameters of the study groups according to the CAC score (n = 187).

	CAC score = 0 (n = 69)	CAC score = 1–400(n = 87)	CAC score > 400(n = 31)	p*	pα	pβ	pγ
Age,years	52.0 ± 9.9	55.2 ± 7.7	57.5 ± 8.6	0.147	-	-	-
Female, n(%)	28 (40.5)	27 (31.0)	9 (29.0)	0.367	-	-	-
Hypertension, n(%)	21 (30.4)	46 (52.8)	18 (58.0)	0.006	0.078	0.028	0.357
Diabetes Mellitus, n(%)	14 (20.2)	23 (26.4)	14 (45.1)	<0.001	<0.001	<0.001	<0.001
Smoking, n(%)	4 (5.7)	4 (4.5)	2 (6.4)	0.896	-	-	-
Total protein, g/dL	70.2 ± 4.4	70.0 ± 4.6	70.5 ± 5.0	0.622	-	-	-
Albumin, mg/dL	4.5 ± 0.2	4.4 ± 0.3	4.3 ± 0.4	0.003	0.007	0.006	0.082
AST, IU/l	23 (19–28)	22 (18–27)	23 (18–26)	0.466	-	-	-
ALT, IU/l	24 (18–36)	24 (19–34)	19 (14–26)	0.033	0.945	0.022	0.012
HbA1c, %	5.8 (5.6–6.2)	5.9 (5.6–6.7)	6.7 (6.1–7.9)	0.010	0.555	0.006	0.007
Glucose, mg/dL	90 (84–106)	94 (87–113)	102 (92–119)	0.220	-	-	-
Creatinine, mg/dL	0.80 ± 0.14	0.86 ± 0.16	0.90 ± 0.14	0.013	0.026	0.006	0.370
Total cholesterol, mg/dL	188.8 ± 46.3	202.8 ± 45.4	174.3 ± 38.2	0.013	0.078	0.140	0.005
LDL-C, mg/dL	112.8 ± 32.3	125.4 ± 37.0	100.3 ± 35.5	0.007	0.030	0.179	0.004
HDL-C, mg/dL	45 (40–54)	44 (38–53)	42 (36–45)	0.029	0.284	0.005	0.076
Triglyceride, mg/dL	123 (104–229)	147 (107–206)	156 (115–218)	0.546	-	-	-
Hemoglobin, g/dL	14.6 ± 1.1	14.3 ± 1.5	14.1 ± 1.6	0.143	-	-	-
WBC,10³/mm³	7.3 ± 2.3	6.9 ± 1.7	7.5 ± 2.0	0.355	-	-	-
Platelet, 10³/mm³	252 (212–304)	256 (230–320)	242 (200–286)	0.161	–	-	-
Neutrophil, 10³/mm³	3.7 (3.1–4.9)	3.9 (3.1–4.8)	4.3 (3.3–5.1)	0.435	-	-	-
Lymphocyte,10³/mm³	2.3 ± 0.9	2.1 ± 0.7	2.1 ± 0.7	0.605	-	-	-
CRP, mg/dL	2.8 (1.3–4.0)	3.1 (1.7–6.2)	7.0 (4.1–12.0)	<0.001	0.017	<0.001	<0.001
LVEF, %	60.4 ± 5.4	65.5 ± 5.8	60.2 ± 6.0	0.084	-	-	-
CAC Score	0 (0–0)	84 (25–176)	811 (631–1385)	<0.001	<0.001	<0.001	<0.001
CAD-RADS Score	0 (0–1)	2 (1–3)	4 (3–5)	<0.001	<0.001	<0.001	<0.001
CAR	0.61 (0.25–0.95)	0.71 (0.38–1.37)	1.57 (0.92–3.07)	<0.001	0.020	<0.001	<0.001

Data are given as mean ± standard deviation , n (%) or median (interquartile range).ALT, Alanine aminotransferase ;AST,* p value between all groups .α p value between CAC Score = 0 and CAC Score = 1–400 groups.β p value between CAC Score = 0 and CAC Score > 400 groups.γ p value between CAC Score = 1–400 and CAC Score > 400 group.

**Table 3:** Baseline characteristics and laboratory parameters of the study groups according to the CAD-RADS Score (n = 187).

Parameters	CAD-RADS score < 4(n = 154)	CAD-RADS score ≥ 4(n = 33)	p
Age,years	51.8 ± 8.2	56.1 ± 7.5	0.214
Female, n(%)	53 (34.4)	11 (33.3)	0.905
Hypertension, n(%)	69 (44.8)	16 (48.4)	0.700
Diabetes Mellitus, n(%)	38 (24.6)	13 (39.3)	0.085
Smoking, n(%)	6 (4.8)	4 (12.1)	0.060
Total protein, g/dL	70.1 ± 4.5	69.6 ± 4.4	0.386
Albumin, mg/dL	4.5 ± 0.3	4.2 ± 0.3	<0.001
AST	23 (19–27)	21 (18–26)	0.443
ALT	24 (18–34)	23 (16–30)	0.202
HbA1c	5.9 (5.6–6.7)	6.7 (6.2–8.0)	0.002
Glucose, mg/dL	92 (84–106)	105 (95–130)	0.001
Creatinine, mg/dL	0.84 ± 0.14	0.87 ± 0.19	0.379
Total cholesterol, mg/dL	188.8 ± 46.3	192.0 ± 52.1	0.771
LDL-C, mg/dL	112.8 ± 32.3	116.7 ± 44.3	0.743
HDL-C, mg/dL	45 (40–54)	41 (31–47)	0.004
Triglyceride, mg/dL	123 (104–229)	173 (101–261)	0.261
Hemoglobin, g/dL	14.5 ± 1.4	13.8 ± 1.5	0.006
WBC,10³/mm³	7.0 ± 1.9	7.8 ± 2.2	0.063
Platelet, 10³/mm³	252 (223–306)	243 (208–307)	0.685
Neutrophil, 10³/mm³	3.8 (3.1–4.8)	4.3 (3.3–5.0)	0.080
Lymphocyte,10³/mm³	2.1 ± 0.8	2.3 ± 0.7	0.087
CRP, mg/dL	3.0 (1.4–4.9)	7.0 (4.1–13.2)	<0.001
LVEF, %	61.0 ± 5.1	60.5 ± 5.7	0.115
CAC Score	10 (0–107)	477 (110–1111)	<0.001
CAR	0.66 (0.30–1.09)	1.73 (1.01–3.33)	<0.001

Data are given as mean ± standard deviation , n (%) or median (interquartile range).ALT, Alanine aminotransferase ;AST, Aspartate aminotransferase; CAC, coronary artery calcium; CAD-RADS, Coronary Artery Disease–Reporting and Data System; CAR, C-reactive protein to albumin ratio; CRP, C-reactive protein; HbA1c, Hemoglobin A1c; HDL-C, high-density lipoprotein cholesterol; LDL-C, low-density lipoprotein cholesterol; LVEF, left ventricular ejection fraction; WBC, white blood cell.

In univariate logistic regression analysis, alanine aminotransferase, HbA1c, creatinine, LDL-C, CAD-RADS score, and CAR were significantly associated with CAC score. Multivariate logistic regression analyses showed that LDL-C, CAD-RADS score, and CAR were independent predictors of CAC score (p < 0.05) (Table 4).

**Table 4:** Univariate and multivariate binary logistic regression analysis for CAC score.

	Univariable analysis		Multivariable analysis	
Variables	OR (95%CI)	p	OR (95%CI)	p
Hypertension	1.839 (0.843–4.015)	0.126	-	-
ALT	0.964 (0.929–1.000)	0.048	0.989 (0.923–1.059)	0.746
HbA1c	1.479 (1.023–2.140)	0.038	1.267 (0.576–2.789)	0.556
Creatinine	17.033 (1.231–235.628)	0.034	7.220 (0.040–1298.843)	0.456
LDL-C	0.983 (0.971–0.996)	0.008	0.963 (0.936–0.991)	0.010
CAD-RADS Score	3.116 (2.102–4.621)	<0.001	3.347 (1.490–7.515)	0.003
CAR	136.239 (6.678–2779.439)	0.001	149.735 (7.468–3002.257)	0.001

ALT, Alanine aminotransferase; CAC, coronary artery calcium; CAD-RADS, Coronary Artery Disease–Reporting and Data System; CAR, C-reactive protein to albumin ratio; CI, Confidence interval; HbA1c, Hemoglobin A1c; LDL-C, low-density lipoprotein cholesterol; OR, odds ratio.

## 4. Discussion

In this study, we showed that patients with the higher CAR values have the higher CAC score and CAD-RADS scores. Our study is the first report in the literature to demonstrate the relationship between CAR and CAC and CAD-RADS scores. Also, we found that CAR, CAD-RADS score, LDL-C were independently associated with CAC score after adjusting for other risk factors. There was a positive correlation between CAR and CAC and CAD-RADS scores. Inflammation is associated with the atherosclerotic process and plays an important role in CAD [18, 19]. Oxidative stress causes atherogenesis by increasing free radical formation and lipid peroxidation. In this way, the severity of vascular inflammation increases [20]. Thus, inflammation and oxidative stress play major role of atherosclerosis and have important effects on the initiation, progression and rupture of lipid-rich lesions [21]. CRP is part of the inflammatory process and is an important indicator of increased inflammation. CRP accumulation has been shown in early atherosclerotic lesions. Also, CRP is chemotactic within white blood cells that have the CRP receptor [22]. In this way, it is thought that atherogenesis increases as a result of the inflammatory process in which monocytes participate. CRP has been shown to affect cultured endothelial cells resulting in decreased nitric oxide and increased endothelin-1 release [23, 24]. The risk of CAD increases in long-term follow-ups in people with higher CRP levels compared to the healthy population [25]. Quaglia et al. showed that plasma CRP is independently associated with coronary atherosclerosis burden [26]. In addition, the trend of increasing CRP after acute coronary syndrome is associated with cardiovascular and all-cause mortality [27].

Serum albumin has been accepted as both a predictor for nutritional assessment and a biomarker of immunocompetence status. However, in the case of persistent systemic inflammation, the serum albumin level decreases, indicating that albumin has a limited role in showing nutritional status [28]. Low serum albumin level is related with disturbanced endothelial function, increased blood viscosity, platelet aggregation, and platelet-induced coronary artery narrowing [29]. Oduncu et al. examined the prognostic utility of albumin in ST-segment elevation myocardial infarction and showed that low serum albumin level was an independent predictor of long-term mortality and development of advanced heart failure [30]. Kurtul et al. showed that serum albumin level in the admission was inversely related to CAD severity in patients with acute coronary syndrome [31]. 

Vascular calcification was regarded as an end result of aging, and the development of CAC was considered a passive consequence of this process. CAC development appears to be an active pathogenic process that is no longer inevitable, and the mechanisms underlying vascular calcification have been identified. Ectopic bone formation, a common feature of atherosclerosis, has been suggested as the basis for CAC [32, 33]. Inflammatory, developmental, and metabolic factors affect all this process. Inflammation in the arterial wall spread by apolipoproteins and oxidized phospholipids is critical for the development of both atherosclerosis and vascular calcification. Several mediators associated with oxidative stress are related to calcification, and there may be an important association between oxidative stress, inflammation, and vascular calcification [34]. The increased prognostic value of CAC has been shown in many studies. Shaw et al. found that CAC has increased information to traditional risk factor assessment in predicting all-cause mortality in 10,377 asymptomatic individuals [35]. CAD-RADS contains current recommendations for CCTA reading. Most of the studies evaluating the prognostic value of CCTA have used an approach focused on stenosis severity, which is the main component of CAD-RADS [36, 37]. van Rosendael et al observed that CTA risk score adding coronary plaque extent, location, severity, and composition enhanced prediction of events compared with the CAD-RADS based on stenosis severity and demonstrated the good prognostic accuracy in an external validation cohort [37]. The novel parameter, CAR is believed to be a more definite indicator of the inflammatory status than CRP or albumin alone. Additionally, CAR was associated with worse prognosis in patients with critical illness and malignancy [38]. There are several studies showed the relationship between CAR and CAD [13, 14, 39, 40]. Calik et al. showed that preprocedural CAR is predicting  in-stent restenosis in patients undergoing successful iliac artery stent implantation [41]. Also, Cinar et al. found that CAR is an independent predictor of all-cause mortality in patients with ST elevation myocardial infarction [42]. Dereli et al. observed that CAR is an independent predictor of the presence of coronary artery ectasia and is significantly correlated with the severity of coronary artery ectasia [43]. Acet et al. found that combination of CAR and GRACE score was an independent predictor of short-term major adverse cardiac events in STEMI patients undergoing PCI [44].

In the light of these studies, we also found a significant relationship between CAR and CAC and between CAR and CAD-RADS. In other words, the close relationship between atherosclerosis and CAR was demonstrated in our study. We claimed that higher CAR can be a predictive marker of atherosclerosis and coronary artery diseses. Furthermore CRP and serum albumin can be used in follow-up and determining the prediction of the CAC score and atherosclerosis. On the other hand, to clear of these hypothesis, further prospective and large studies are needed. 

There are several limitations of this study. Firstly, this study was retrospective. Secondly, this study has limited number of participants. Using a spot laboratory value rather than values at a time-interval is another limitation of the study. Nevertheless, we could not evaluate other cytokines or inflammatory markers, either. 

In conclusion, our study is the first paper about the relationship between CAR and CAC score and CAD-RADS score. The result may be clinically useful because CAR is easily accessible, inexpensive method that can be considered. CAR may be a marker used in the management of patients before invasive coronary angiography. Further studies are needed to clarify the pathophysiologic role of CAR in patients with atherosclerotic coronary heart disease.

## Author contributions

MAE and KGY contributed to: (1) substantial contributions to design and conception, or obtaining of data, or analysis and interpretation of data, (2) drafting the article or revising it critically for important intellectual content, and (3) final approval of the version to be published. 

## Informed consent

Ethics committee approval was received for this study from University of Health Sciences, Ankara City Hospital (Ethics committee no: E1-21-1567). Consent form from patients was not received as this is a retrospective study.
